# Wound Healing in a Patient with Psoriasis Vulgaris and Femur Megaprosthesis Implantation

**DOI:** 10.1155/2008/509242

**Published:** 2008-04-13

**Authors:** Markus Nottrott, Jendrik Hardes, Winfried Winkelmann, Georg Gosheger

**Affiliations:** ^1^Department of Orthopaedic Surgery, Haukeland University Hospital, N-5021 Bergen, Norway; ^2^Department of Orthopaedics, Münster University Hospital, D-48149 Münster, Germany

## Abstract

Extraskeletal mesenchymal chondrosarcoma is extremely rare and, in combination with psoriasis, it has never been described before. We report a case of wide resection of an extraskeletal chondrosarcoma of the thigh and reconstruction with a femoral megaprosthesis in a patient with psoriasis vulgaris. Special emphasis has been laid to postoperative wound healing in psoriatic skin which did not show any problems.

## 1. INTRODUCTION

Psoriasis in patients who should undergo surgery is a controversially discussed problem and today there are no controlled, prospective studies existing on this topic [1]. While elective surgery in psoriatic skin is a more common problem, surgery in psoriatic skin for implantation of a megaprosthesis because of a chondrosarcoma has never been described before. We report a case of the resection of an extraskeletal chondrosarcoma of the thigh and reconstruction with a femoral megaprosthesis in a patient with psoriasis vulgaris. Special attention is given to postoperative wound healing in psoriatic skin.

## 2. CASE REPORT

A 20-year-old man with a history of Psoriasis vulgaris was presented to our department for resection of an extraosseous mesenchymal chondrosarcoma of the left thigh.

The patient recognized a half year before admission a tumour in his left thigh and suffered of pain over some weeks before he contacted a local hospital where X-rays and an MRI were suspicious for a malignant tumour. Physical examination showed no skin disorders at this point of time. Open biopsy showed an extraosseous mesenchymal chondrosarcoma G3. Tumour size was 18 × 8 × 14 cm. The tumour was localized close to the femur in the middle third of the thigh. Tumour staging with CT of the chest showed no pathology in the thorax but abdominal CT revealed two intraabdominal lymph node metastases besides the left A. and V. iliaca interna. Skeletal scintigraphy supported these findings with high uptake in the middle third of the thigh and the two lymph nodes.

Chemotherapy with Ifosfamide and Adriamycine was initiated with a good initial tumour response regarding tumour size and lymph node reduction. Four months later, a left inguinal and iliacal lymphadenectomy was performed where six metastases were removed in another hospital. Chemotherapy was continued afterwards with a total number of 5 cycles with Adriamycine (60 mg/m^2^) and Ifosfamide (8 g/m^2^) up to 5 months after diagnosis. Curative treatment with a wide tumour resection of all contaminated tissue, including the groin (high amputation), was planned but preoperative restaging one month later showed bipulmonal and new inguinal metastases in CT and tumour growth in the thigh. Therefore, curative treatment seemed to be impossible and it was decided to perform a marginal resection of the tumour and reconstruction of the defect with a cemented diaphyseal femurprosthesis implantation allowing immediate weight bearing and mobilization of the patient.

**Figure 1:**
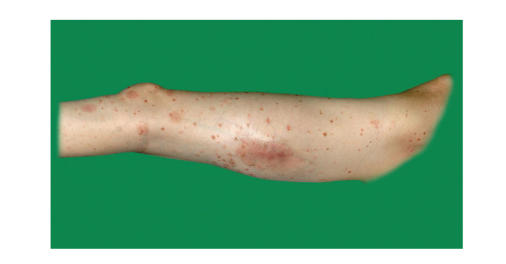
Preoperative clinical picture showing psoriasis vulgaris efflorescence's on the left thigh.

**Figure 2:**
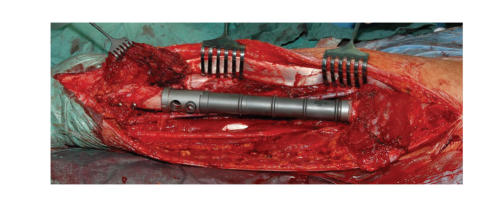
Left thigh after wide tumour resection and implantation of a Mutars femur diaphysis implant.

**Figure 3:**
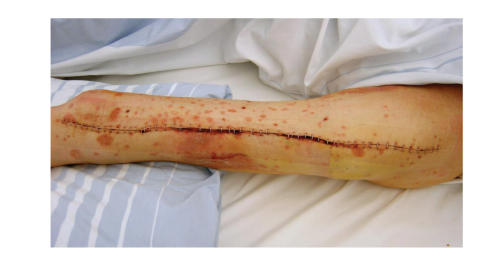
Left thigh 1 week postoperatively with a slight necrosis of the skin in central areas of the wound.

**Figure 4:**
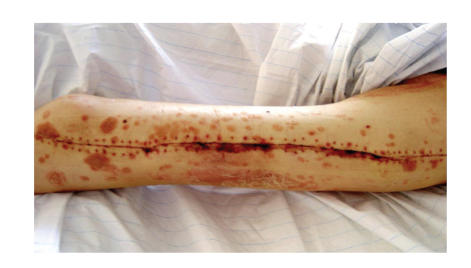
Left thigh 2 weeks postoperatively after removal of the clips.

Also at this restaging examination the patient had no skin disorders, but on admission to the hospital two weeks later he presented with ubiquitary psoriasis vulgaris efflorescence's especially on the left thigh. These lesions were characterized as erythemato-squamouse. Treatment with Clobetasol-17-propionat (Dermoxin) crème was initiated and the patient responded well to this treatment (Figure 1). Because of tumour progression and in spite of an expected increased postoperatively infection risk, operation with local wide tumour resection (360 mm) and implantation of a Mutars femur diaphyseal implant was performed one week after psoriasis treatment started (Figure 2). Intraoperatively, subcutaneous dissection appeared to be difficult because of the psoriasis efflorescence's effect on the subcutaneous tissue. However, closure of the subcutaneous and cutaneous tissue was without any problems by using an M. biceps femoris flap covering the prosthesis. Postoperatively the patient received Ceftriaxone (Rocephin) 2 g × 1 and Clindamicine (Sobelin) 600 mg × 3 intra venously for one week. Wound healing did not show any problems. No wound infection was seen but a slight necrosis of the skin in central areas of the wound without any clinically relevance and without any relationship to the psoriasis efflorescence's (Figure 3) was observed. Psoriasis treatment was changed from Clobetasol-17-propionate crème to Calcipotriol Betamethasone (Psorcutan) crème and clinically a slight remission of the efflorescence's could be observed (Figure 4). Two weeks postoperatively, the patient was mobilized and dismissed from the hospital. Histological examination of the tumour showed an extraskeletal mesenchymal chondrosarcoma G3 with a small-blue-round cell component. Resection margins were wide and the response grade was V according to Salzer-Kuntschik [2]. Palliative chemotherapy with Topotecan/Cyclophosphamide was initiated. Treatment with Psorcutan crème of the psoriasis efflorescence's was continued.

## 3. DISCUSSION

Extrasceletal mesenchymal chondrosarcoma is extremely rare [3] and in combination with psoriasis it is even more rare. To our knowledge the combination of psoriasis vulgaris and this tumour entity has never been reported before. Furthermore, there have been no reports in the literature describing wound healing of more than 30 cm in psoriatic skin. Therefore, we had difficulties in decision making to operate or not with respect to concerns about wound healing and sepsis.

While some publications indicate that there is no increased risk for infection or wound healing disturbances after elective surgery in psoriatic skin [4] and psoriasis is not an absolute contraindication to surgery [5], others state that they have seen more postoperative sepsis in psoriatic patients [6]. The decision to perform an operative procedure with limb salvage that usually is conducted to a high postoperative infection rate without any extra skin disorder was done to persuade the patient to have a good rest life quality. In case of a postoperative deep wound infection or widespread skin necrosis, an amputation would have been performed. In this case, preoperative treatment of the psoriatic skin was limited by the necessity to perform the operation as soon as possible with respect to rapid tumour growth.

After operation the patient received a prolonged antibiotic prophylaxis over one week and we could not observe any wound infection or healing complications related to the psoriatic skin. A slight necrosis of the skin in central parts of the wound is not unusual in such operations with wide subcutaneous preparation and has no clinical relevance that causes an intervention.

Our case shows that megaprosthesis implantation is possible in patients with psoriatic lesions in the operation field. There should be applied an adequate psoriasis treatment preoperatively if possible and a prolonged antibiotic prophylaxis seems to be helpful. Whether there is an increased infection risk or not remains unclear.
